# Contrasting allelic effects for pistachio salinity tolerance in juvenile and mature trees

**DOI:** 10.1038/s41598-023-41195-1

**Published:** 2023-09-01

**Authors:** Abdollatif Sheikhi, Mohammad M. Arab, Matthew Davis, William J. Palmer, Richard Michelmore, Pat J. Brown

**Affiliations:** 1 Department of Plant Sciences, University of California, Davis, USA; 2 The Genome Center, University of California, Davis, USA; 3 Present Address: Gencove Inc, New York, USA

**Keywords:** Plant genetics, Plant breeding

## Abstract

Breeding perennial tree crops often requires prediction of mature performance from juvenile data. To assess the utility of juvenile screens to predict salinity tolerance of mature pistachio trees, we compared performance of 3-month ungrafted seedlings and 4-year-old grafted rootstocks under salinity stress. The QTL allele associated with higher salt exclusion from seedling leaves conferred lower growth in saline field conditions, suggesting that mapping QTL in seedlings may be easier than discerning the optimal allele for field performance.

## Introduction

Global food production relies on groundwater sources that are becoming increasingly saline due to unsustainable irrigation demands. Pistachio (*Pistacia vera* L*.)* is among the most salt tolerant of tree crops^1^ and has been widely planted in marginal, saline soils in the southwestern US, the Near East, and the Mediterranean basin. However, chronic overuse of groundwater is now impacting pistachio yields in the world’s top two pistachio-producing regions, California and Iran. In California, which accounts for over one quarter of global production, total annual agricultural losses due to increased soil salinity are estimated at $3.7B, approximately 10% of total agricultural revenue^2^. The Rafsanjan province of Iran formerly produced over 20% of the global pistachio supply, but pistachio acreage has decreased nearly 30% since 2006 (from 110,000 to 80,000 ha) due to increasing soil salinity and decreasing groundwater volume^3^.

Rootstock breeding provides opportunities to improve biotic and abiotic stress resistance in tree crops without sacrificing yield potential and quality characteristics in the scion^4^. Pistachio rootstocks in the US, and increasingly in other countries, are largely derived from the UCB-1 population, F_1_ seedlings from a single interspecific *Pistacia atlantica* X *P. integerrima* cross. *P. atlantica* is thought to provide resistance to cold, nematodes, and salt, whereas *P. integerrima* provides resistance to *Verticillium dahliae*^5^. The UCB-1 population is highly polymorphic and segregates for several large-effect QTLs for vigor^6,10^. Previous studies indicate that UCB-1 individuals are more salt-tolerant than the *P. integerrima* parent^7^. Proposed mechanisms for sodium tolerance in UCB-1 include sequestration in vacuoles of the root cortex^8^, exclusion through increased suberization of the endodermis^8^, and active unloading of sodium from the phloem^9^. However, genetic variation for salt tolerance in the UCB-1 population has not previously been explored.

Genetic improvement of trees is hampered by long generation times, space constraints on population size, lower availability of genetic resources including reference assemblies and mapping populations, and high heterozygosity of available germplasm^10^: progeny of an inter-specific cross between two obligatorily outcrossing and therefore heterozygous parents such as the UCB-1 population segregate for four parental haplotypes. Taking advantage of genome assemblies recently generated for *P. atlantica* and *P. integerrima*^11^ as well as the high degree of divergence between these species, we genotyped UCB-1 seedlings using reduced-representation Illumina sequencing and aligned reads from these interspecific hybrids to both parental genomes simultaneously. This novel method generates haploid genotype calls, uses all the sequence data without depth thresholding, and leads to higher concordance between replicated genotypes (Brown 2022 submitted). Three experiments were then performed to assess salinity tolerance of pistachio UCB-1 individuals both as juvenile ungrafted seedlings and as rootstocks of grafted mature trees in a 4-year-old orchard.

## Results and discussion

**Figure 1:**
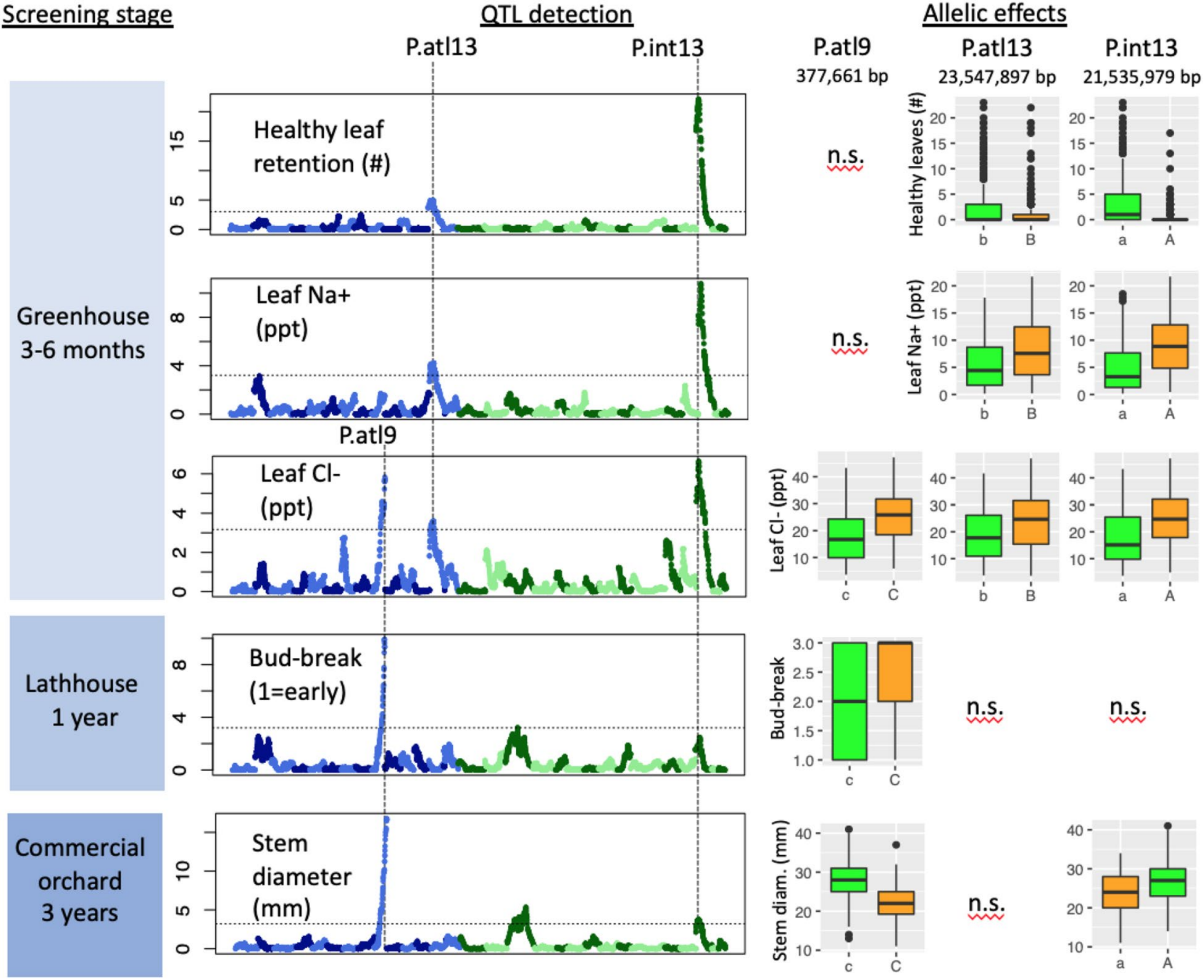
QTL detection and allelic effects for five traits measured over different stages of screening in the greenhouse, lathhouse, and a commercial orchard. Manhattan plots display *P. atlantica* and *P. integerrima* chromosomes alternating in light and dark blue and green, respectively with dashed horizontal lines indicating alpha = 0.05 significance thresholds. Y axis units are LOD scores; X axis units are cM. Three QTL are highlighted and named after the chromosome on which they appear: P.atl9, P.atl13, and P.int13. Boxplots show allelic effects for significant QTL with beneficial and deleterious alleles colored green and orange, respectively. Boxplots use the most significant SNP at each QTL (for stem diameter at P.atl9; for healthy leaf retention at P.atl13 and P.int13) to enable direct comparison of allelic effects between traits. Uppercase and lowercase letters represent the major and minor alleles at each QTL, but in all three cases allele frequencies are very close to 0.5.

First, a greenhouse salt screen of 768 2-month-old seedlings was conducted. Seedlings were subjected to increasingly saline conditions (50–200 mM NaCl) in the greenhouse, and then phenotyped for healthy leaf retention (HLR) as well as leaf sodium (Na+) and chloride (Cl−) content after 4 months of salinity treatment. Linear regression identified two major loci affecting all three phenotypes (HLR, Na+, and Cl−), on chromosomes 13 of *P. atlantica* and *P. integerrima,* respectively (P.atl13 and P.int13 in Fig. 1; alpha < 0.05 using 1000 permutations; see Supplementary Table 6 for LOD scores and QTL confidence intervals). At both loci, alleles associated with higher HLR were associated with lower Na+ and Cl−. Seedlings carrying both high salt alleles (A/B) retained an average of five fewer healthy leaves, 6.3 parts per thousand (ppt) higher leaf Na+, and 10.7 ppt higher leaf Cl− than seedlings carrying both low salt alleles (a/b). High salt alleles also significantly increased leaf levels of boron and lithium, and significantly decreased leaf levels of potassium and rubidium (Supplementary Fig. S1).

Second, 305 1-year-old UCB-1 seedlings were phenotyped for bud-break (scored categorically as early, middle, or late) as they came out of dormancy in a lath-house before planting into an orchard on saline soil (soils tests averaged 3, 6, and 10 dS/m at depths of 0–2, 2–4, and 4–6″, respectively; Supplementary Table S1), where they were grafted with a standard scion and subjected to the same management practices as the rest of the commercial orchard. After 3 years of growth, stem diameter was measured. Surprisingly, QTL alleles associated with higher leaf retention and lower leaf salt content in the greenhouse screen (a/b) were associated with smaller stem diameter under field conditions. The allelic effects panel of Fig. 1 shows that the beneficial (green) and deleterious (orange) alleles at P.int13 are reversed in data from 4-year-old trees in commercial orchards compared to 6-month-old trees in the greenhouse. Both QTL for salt tolerance had relatively minor effects on stem diameter (r^2^ = 0.06 for P.int13; P.atl13 not significant at p < 0.05) compared to a previously-reported^6,11^ QTL for stem diameter on chromosome 9, P.atl9 (r^2^ = 0.23 in our study), which co-localized precisely with QTL for bud-break (r^2^ = 0.06) and leaf chloride (r^2^ = 0.13) in the lath-house and greenhouse, respectively. The allele associated with early bud-break in ungrafted rootstocks is associated with higher stem diameter in grafted commercial orchards throughout California^6,11^, suggesting that differences in rootstock phenology can affect size and yield of the grafted scion, perhaps due to heterochrony in carbohydrate metabolism. This correlation between scion growth and rootstock phenology is tantalizing, since phenology can be measured with high heritability in juvenile, ungrafted trees, while stem diameter in juvenile trees is a poor predictor of vigor at maturity^6^.

**Figure 2:**
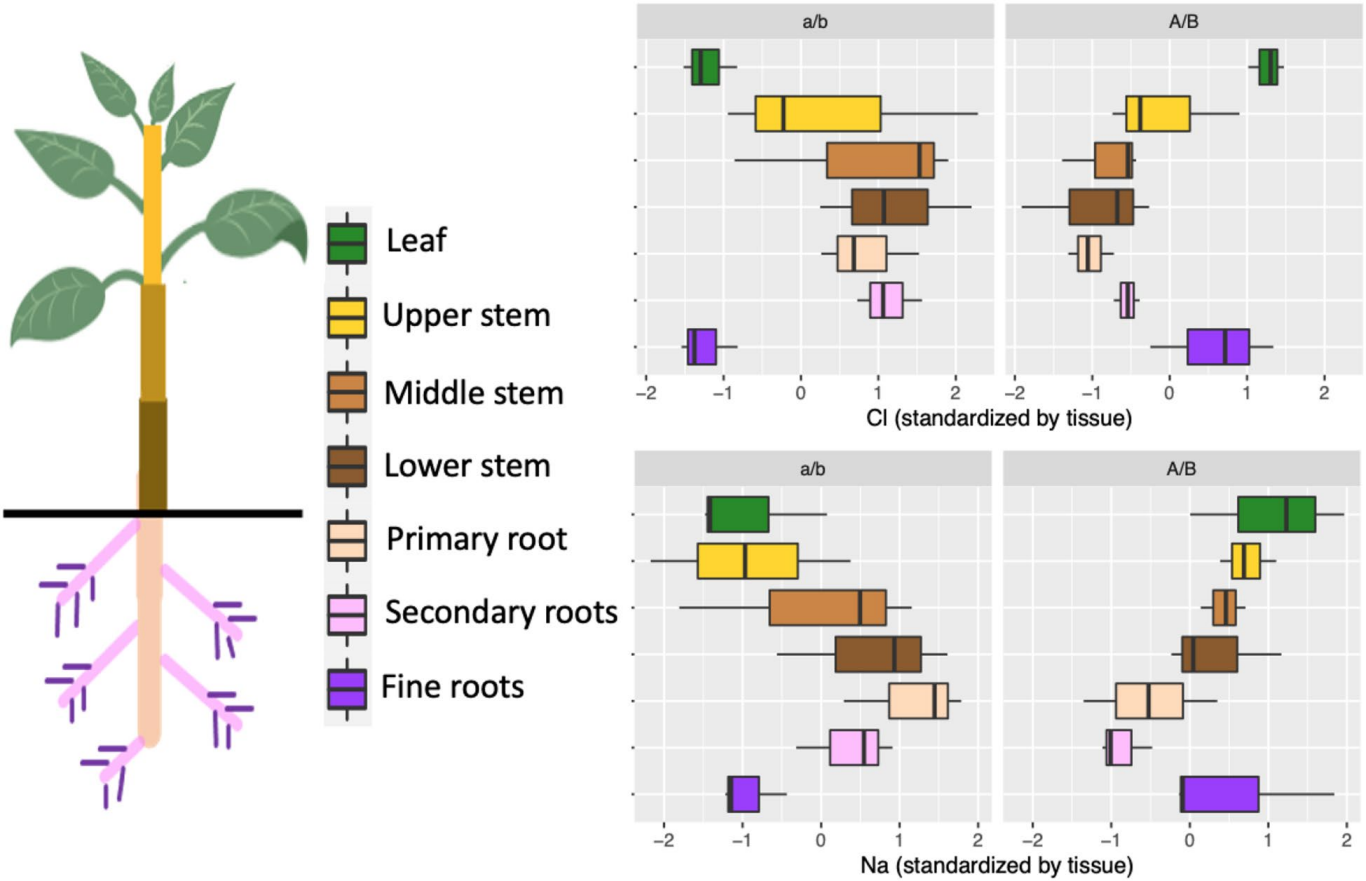
Effects of P.int13/P.atl13 haplotype (a/b versus A/B) on standardized Na+ and Cl− concentrations across 7 tissue types. The a/b haplotype associated with low leaf salt and superior leaf retention in a short-term greenhouse screen has higher levels of salt in woody tissues and lower long-term growth in the orchard.

Third, to investigate whether allelic differences in leaf salt accumulation were due to exclusion or sequestration, and to investigate reasons for the surprising result of the beneficial allele at P.int13 switching in the second experiment, we conducted another greenhouse salt screen using three seedlings of each of the four QTL haplotypes (a/b, a/B, A/b, and A/B; 12 seedlings total) and quantified Na+ and Cl− content across seven tissue types (Fig. 2; all four haplotypes are shown in Supplementary Fig. S2). QTL alleles associated with reduced Na+ and Cl− content in leaves were associated with elevated Na+ and Cl− content in woody tissues of the shoot and root, suggesting that allelic differences are due primarily to sequestration^8,9^ rather than exclusion. To ease comparison of allelic effects between tissues, Fig. 2 shows salt concentrations normalized within tissues; for raw data see Supplementary Fig. S3. Additionally, the molar ratio of Na+ to Cl− is higher in woody tissues than in leaves and fine roots and decreases towards the top of the stem, supporting the model of active Na+ retrieval from the phloem previously proposed^9^ (Supplementary Fig. S4). Given that the P.int13 allele associated with higher salt in wood is also associated with reduced long-term field growth, we infer that salt sequestration in woody tissues may impose a long-term fitness penalty that is not apparent in short-term screens. In deciduous trees, salt movement to leaves with the opportunity to shed leaves each year may be a superior strategy to long-term sequestration in woody tissue.

Assessing the true utility of the variants reported here for increasing or maintaining pistachio yields under saline and non-saline conditions will require multi-year, multi-environment yield trials. However, this study highlights the difficulty of extrapolating from results of screens of young plants to predict long-term performance of woody perennials and demonstrates that in the genomic era, our ability to rapidly genetically dissect phenotypes now greatly exceeds our ability to identify the optimal allele for field performance.

## Methods

### Plant materials, growth conditions, and phenotyping

Greenhouse experiments were performed at UC Davis in 2018–2019, and field data were collected from a commercial pistachio orchard near Mendota, CA, USA in September 2021. UCB-1 seeds obtained from Foundation Plant Services (Davis, CA; https://fps.ucdavis.edu/) were submerged in diH_2_0 for 24 h, surface sterilized, and stratified for 6 weeks at 4 °C before germination in 72-cell flats and transplanted into 2 L pots when they had reached 15–20 cm in height. “UC Davis” soil mix was used for both greenhouse experiments. Greenhouse conditions were set to 28/22 °C day/night temperatures and 70–80% relative humidity, with artificial light provided for 12 h/day with incomplete exclusion of natural light. For the first greenhouse experiment, 768 seedlings were grown for 3 months on daily fertigation with half-strength Hoagland's solution before initiation of salinity stress. For the second greenhouse experiment involving sampling from seven tissues, 9-month-old seedlings were used. Salinity treatment was applied using a fully automated injector system (Mixrite, 1–10%) to deliver dilutions of a 5 M NaCl stock solution at a rate of 150 mL/pot/day. Salinity treatments began with 100 mM NaCI on days 1–6, increasing to 150 mM on days 7–66 and finally to 200 mM on days 67–127. At the end of first experiment, each seedling was scored for the number of healthy leaves remaining (HLR; a “healthy” leaf was defined as one with > 50% green photosynthetic tissue). For a random selection of 200 plants, 1–3 leaves from each seedling were pooled, dried overnight at 80C, and ground into powder using a GenoGrinder (Fisher Scientific) before measurement of Na+ and Cl− concentration. In the second experiment, total biomass from each plant was divided amongst seven tissue types (fine roots, secondary roots, primary roots, lower stem, middle stem, upper stem, and leaves) and woody tissues were passed through a knife mill (Wiley) before grinding. Na+ concentration was measured using inductively coupled plasma-mass spectrometry at the ionomics facility of the Donald Danforth Plant Science Center (St. Louis, MO). Cl− concentration was measured by titration using a chloride analyzer (M926, Nelson-Jameson, Marshfield, WI). Bud-break was scored categorically as early/mid/late in 306 1-year-old seedlings in a lath-house. After three seasons of growth in a commercial orchard, stem diameter of the rootstock was measured using digital calipers (iGaging, Anytime Inc.) at 60 cm above soil level. Soil salinity tests in the commercial orchards are shown in Supplementary Table S1, and plant phenotypes are shown in Supplementary Tables S2–3. All methods were performed in accordance with relevant state and federal regulations.

### DNA extraction and genotyping

Tissue samples were collected from young leaves, freeze-dried, and pulverized using GenoGrinder 2000 (Spex CertiPrep Inc. Metuchen, NJ, USA) at 650 strokes/min for 15 s. Genomic DNA was isolated using 96-well silica filter plates (Epoch Life Science #2020-001). DNA was quantified using PicoGreen (Thermo Fisher Scientific, Waltham, MA) on a Synergy™ HT plate reader (Bio-Tek Instruments, Winooski, VT, USA) and diluted to 50 ng/µl in EB buffer. Genomic libraries were constructed using the two-enzyme GBS approach^12^ using simultaneous restriction-ligation with HindIII-HF, MseI, and T4 DNA Ligase (New England Biolabs). Restriction-ligation reactions were pooled by 96-well plate, purified using AMPure XP beads, PCR-amplified using Phusion Master Mix (NEB #M0531), and purified again using AMPure XP beads. A DNA7500 chip in an Agilent 2100 Bioanalyzer was used to determine average library size and concentration, and to dilute each library to 10 nM before submission for sequencing on an Illumina Hiseq 2500 (SR100) at the UC Davis Genome Center (Davis, CA).

### SNP discovery and imputation

The TASSEL GBS pipeline^13^ was used to call SNPs from raw fastq data using BWA^14^ to align 64 bp tags that occurred at least 10 times in the dataset using dual alignment^15^ against the *P. atlantica* and *P. integerrima* genome assemblies^11^ concatenated together, resulting in a dataset of primarily homozygous genotypes. FSFHap^16^ was used for imputation using the Window LD algorithm.

### Genetic map construction, QTL mapping, and statistical analysis

After imputation, we first filtered out taxa, and then sites, with > 5% heterozygous and/or missing values, and a chi-square test in R^17^ was used to test allele frequencies and discard markers that showed severe distortion from the expected 1:1 ratio (p < 0.00001). ASMap^18^ was used to create a genetic map using “doubled haploid” as the population type. The resulting map (Supplementary Table S4) contained 30 linkage groups (15 each from *P. atlantica* and *P. integerrima*), 682 individuals, and 9501 SNPs. Mapping of quantitative trait loci was performed on untransformed phenotypes in R/qtl^19^ using the scanone() function and the default EM algorithm, with significance thresholds established using 1000 permutations. It was not necessary to correct for population structure in this biparental population. LOD scores for all marker-trait combinations are shown in Supplementary Table S5, and significant QTL are shown in Supplementary Table S6.

### Supplementary Information

Supplementary Figures.

Supplementary Tables.

## Data Availability

Raw sequence data for this project have been submitted to the NCBI SRA under BioProject PRJNA909784 (accessions SRX18653732 and SRX18653733). Phenotype data, imputed genotype data, and QTL results are included as supplemental tables.
