# Supplementary material for: Contrasting allelic effects for pistachio salinity tolerance in juvenile and mature trees
Source: Sci Rep. 2023 Sep 1;13:14391. doi: 10.1038/s41598-023-41195-1 (PMC10474094; doi:10.1038/s41598-023-41195-1)
Supplement: Supplementary file 1 — Supplementary Figures. [file 41598_2023_41195_MOESM1_ESM.docx]

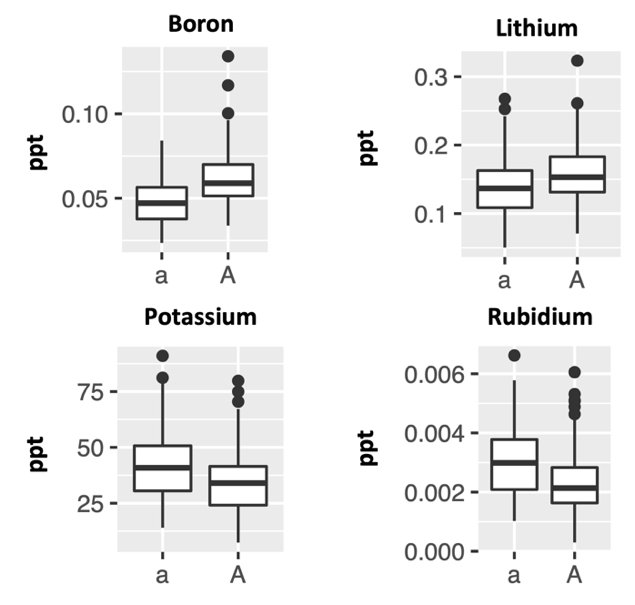


Figure S1. Effects of P.int13 genotype on leaf concentrations (parts per thousand) of Boron, Lithium, Potassium, and Rubidium. All effects are significant at p<0.01.


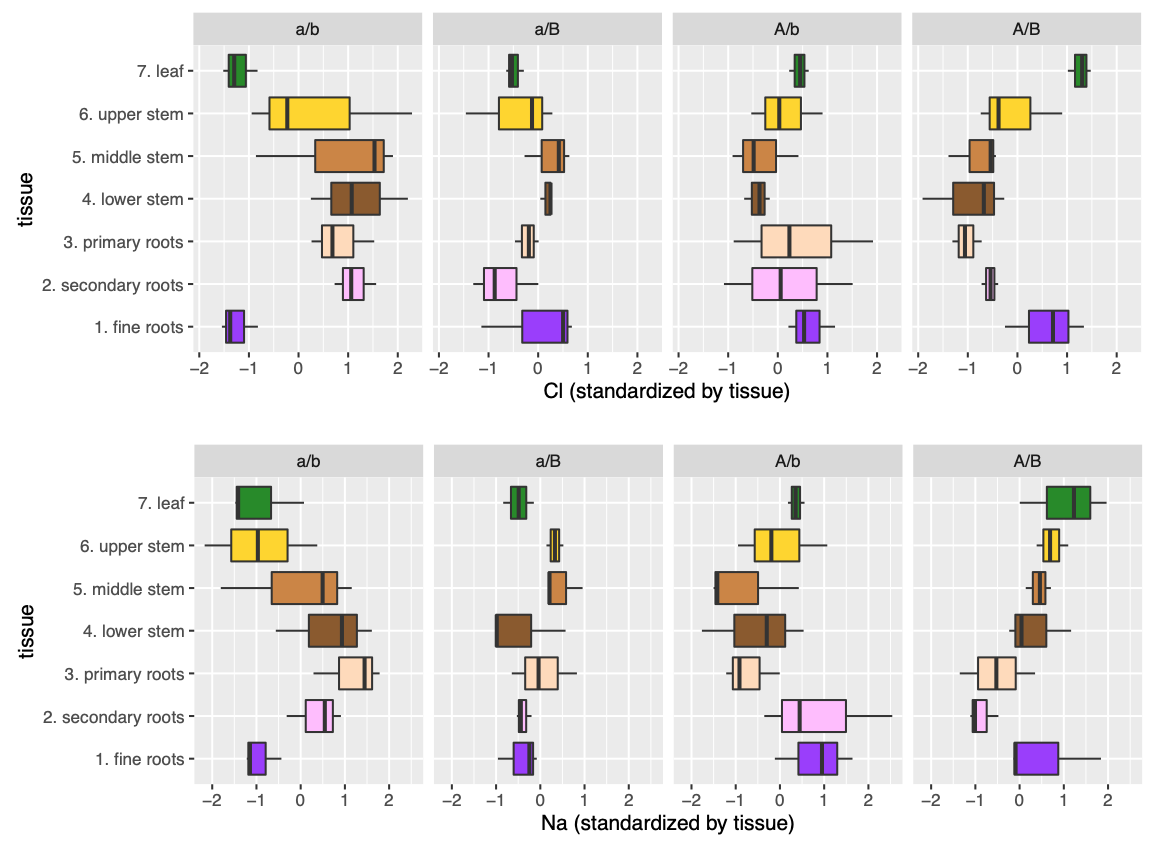


Figure S2. Effects of all four P.int13/P.atl13 haplotypes on standardized Na+ and Cl- concentrations in seven tissue types.


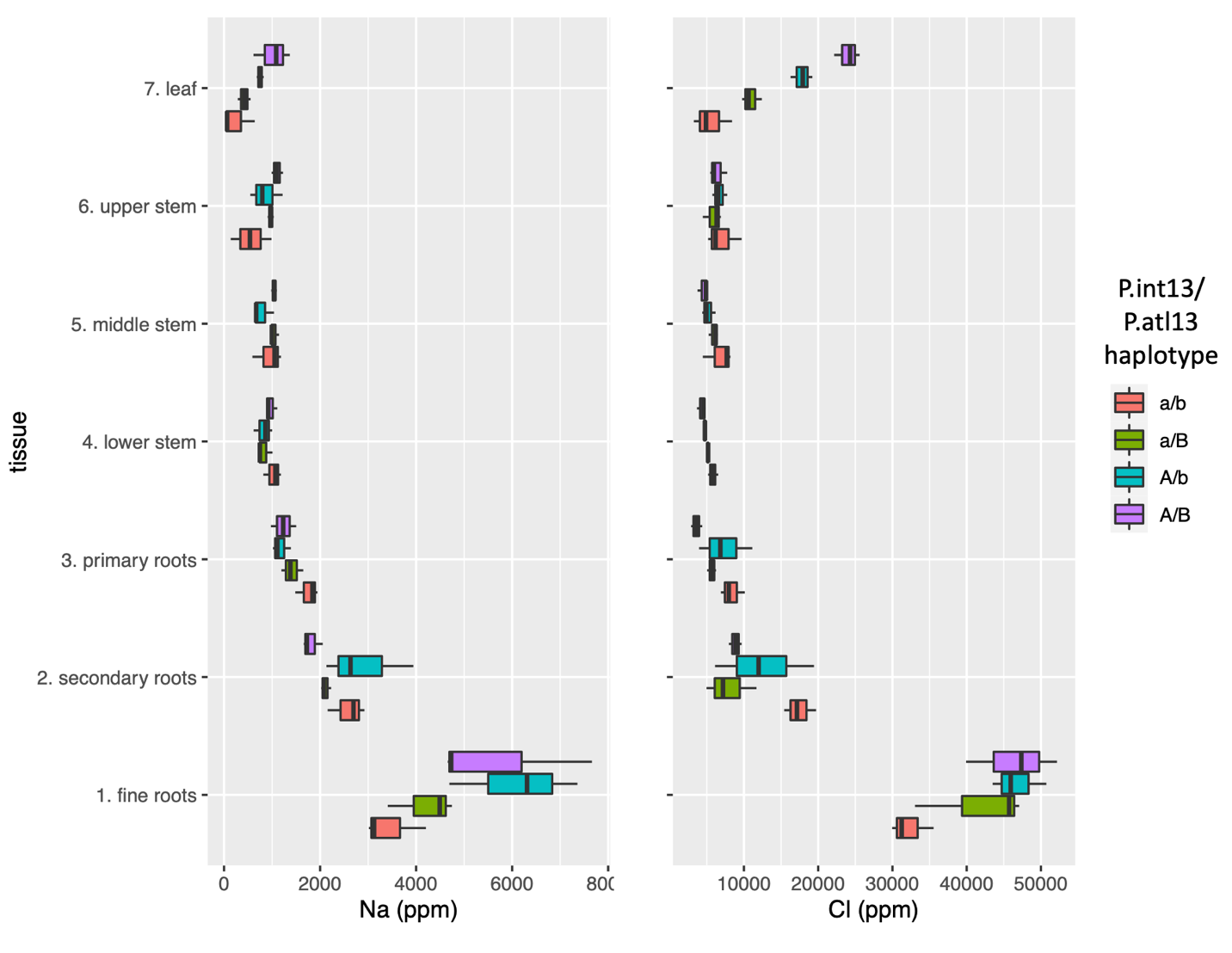


Figure S3. Effects of P.int13/P.atl13 haplotype on raw Na+ and Cl- concentrations in seven tissue types.


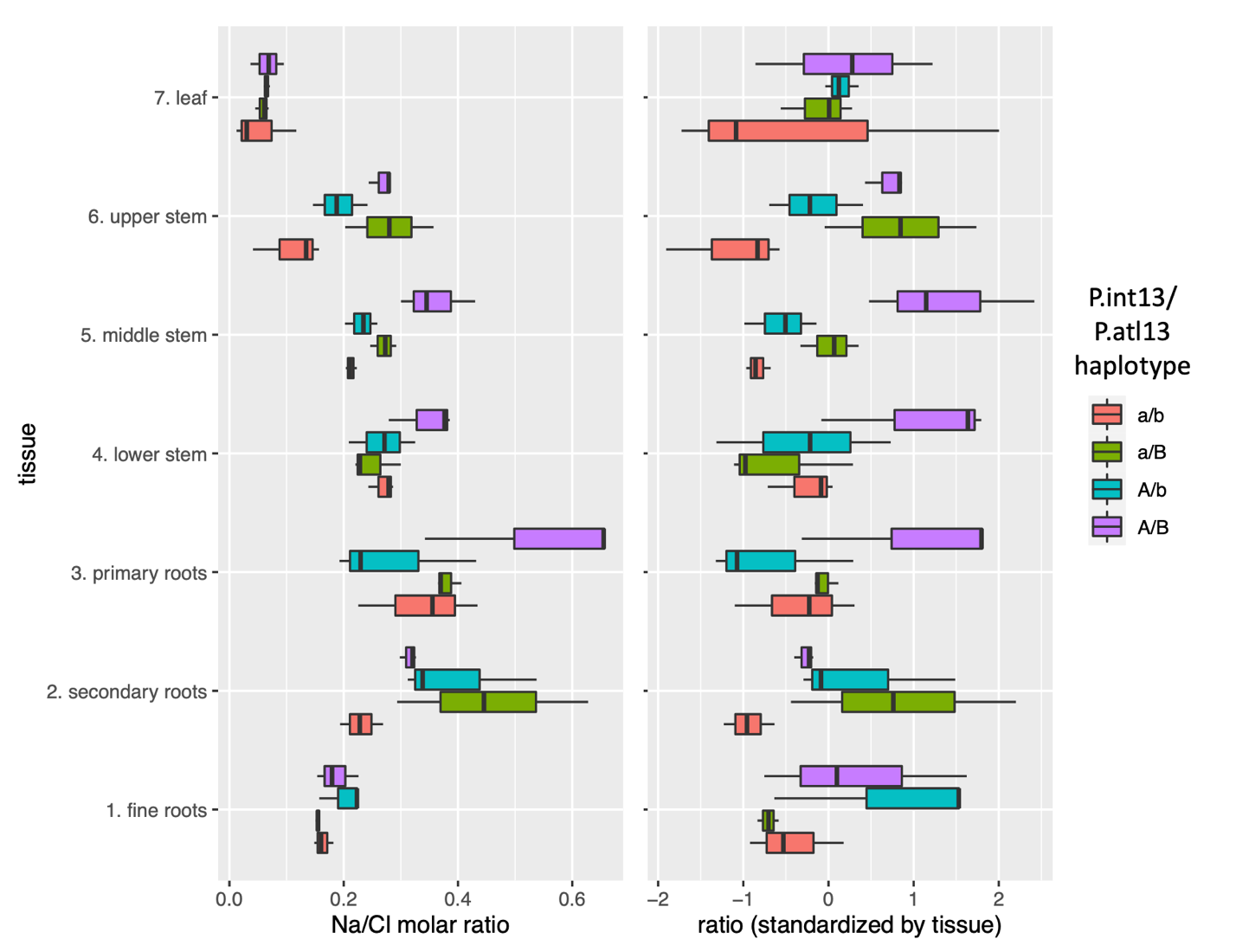


Figure S4. Effects of P.int13/P.atl13 haplotype on raw and standardized Na/Cl molar ratio in seven tissue types.
